# Identification of metabolic biomarkers and therapeutic targets in the thymoma-associated myasthenia gravis treated with methylprednisolone

**DOI:** 10.1007/s12672-025-02700-2

**Published:** 2025-05-26

**Authors:** Shanshan Gu, Xu Wang, Hongxia Yang, Yaxuan Wang, Congya Yan, Xiaoting Lin, Peng Liu, Lu Liu, Li Meng, Guoyan Qi

**Affiliations:** 1 Department of Oncology, Hebei Medical University, Shijiazhuang, China; 2 Department of Radiation Oncology, The Affiliated Tai’an City Central Hospital of Qingdao University, Taian, China; 3 Center of Treatment of Myasthenia Gravis, People’s Hospital of Shijiazhuang Affiliated to Hebei Medical University, Shijiazhuang, China; 4 Hebei Provincial Clinical Research Center for Myasthenia Gravis, Shijiazhuang, China; 5 The Second Affiliated Hospital of Shandong First Medical University, Taian, China

**Keywords:** Thymoma, Myasthenia gravis, Metabolomics, LC–MS, Network pharmacology

## Abstract

**Objective:**

This study aims to screen and identify metabolic biomarkers and targets for methylprednisolone treatment of thymoma with myasthenia gravis (MG) through metabolomics and network pharmacology analysis, thereby improving guidance for clinical medication and treatment.

**Methods:**

Serum from 15 patients with thymoma accompanied by severe MG was collected. Changes in serum metabolite levels before and after methylprednisolone treatment were determined using liquid chromatography-mass spectrometry (LC—MS). The raw mass spectrometry fragment information obtained was integrated and interpreted using the metabolomics data analysis software Progenesis QI v2.3. Differential metabolites were screened and identified using univariate and multivariate statistical analysis methods. Subsequently, potential targets of methylprednisolone treatment were identified through network pharmacology, and the mechanism of action of methylprednisolone in treating thymoma with MG was explored in conjunction with metabolomics. Finally, key targets and the upstream synthetic enzymes of critical metabolites identified were validated using Enzyme-Linked Immunosorbent Assay (ELISA).

**Results:**

A total of 148 differential metabolites were identified in the metabolomics study, among which key metabolites ceramide (Cer) and sphingomyelin (SM) play a significant role in cell immune regulation, inflammatory response, and tumor control. Network pharmacology analysis revealed that tumor necrosis factor (TNF) could serve as a potential target for methylprednisolone treatment of thymoma with MG. ELISA validation results showed that the key target TNF and the upstream synthetic enzymes of the key metabolites SM and Cer were all downregulated after methylprednisolone treatment, with the differences being statistically significant (*P* < 0.05).

**Conclusion:**

Our Study reveals that TNF could serve as a potential target for methylprednisolone treatment of Thymoma-associated MG, and Cer and SM could act as potential metabolic biomarkers to assess its treatment efficacy.

## Introduction

Thymoma is a rare tumor originating in the mediastinum, with an annual incidence of approximately 0.13–0.32 per 100,000 [1]. Epidemiological studies have shown a close association between thymoma and myasthenia gravis (MG). Among patients with thymoma, approximately 15–20% also have concurrent MG [2]. Thymoma may contribute to the development of MG by affecting the thymic microenvironment, promoting the survival and proliferation of autoreactive T cells, and generating autoimmune antibodies [3]. MG is a neuromuscular disease closely related to autoimmunity, with a global annual incidence of approximately 1.7–21.3 per million [4]. Clinically, MG patients often present with fluctuating muscle weakness, which typically starts with involvement of the ocular muscles. In severe cases, the respiratory muscles may also be affected, leading to respiratory failure and death [5]. Thymic abnormalities are very common in MG patients, with thymomas accounting for approximately 15% of cases [6].

For patients with Thymoma-associated Myasthenia Gravis (TMG), the majority initially undergo surgical treatment to remove the thymoma, which can improve clinical symptoms in some patients [7]. However, surgery alone often does not fully alleviate the symptoms of MG, and thymoma may recur after surgery, possibly related to the residual thymoma cells in the body or"ectopic thymus"in the anterior mediastinum. Currently, for patients with persistent muscle weakness symptoms after thymectomy, the primary treatment method is glucocorticoid therapy, which can quickly alleviate the symptoms of muscle weakness, also inhibit the growth and recurrence of residual thymoma, and promote tumor cell apoptosis [8], and is recognized by most clinicians.

Glucocorticoids (GC) are a type of steroid hormone with potent anti-inflammatory, anti-allergic, and immunosuppressive effects, often used in the treatment of autoimmune diseases. In addition, GC has an inhibitory effect on some tumors. Studies have found that GC can inhibit the synthesis and release of prostaglandins in prostate cancer cells, delaying tumor cell growth and spread [9]. In cell experiments, GC can inhibit the colony formation, migration, and growth of the pancreatic cancer cell line PANC-1 [10]. Methylprednisolone, a commonly used exogenous medium-effect glucocorticoid in clinical practice, has stronger anti-inflammatory and immunosuppressive effects compared to its counterpart, prednisolone.

Although previous studies have revealed the association between thymoma and MG, the pathogenesis of TMG is complex, involving autoimmune responses and tumor biology [11]. Currently, there is a lack of systematic research on specific targets and metabolic biomarkers for GC, especially methylprednisolone, in the treatment of TMG.In clinical practice, it has been observed that due to the non-specificity of GC targets, methylprednisolone exerts broad therapeutic effects but also causes various adverse reactions, such as hyperlipidemia, hyperglycemia, and osteoporosis. These adverse effects are primarily attributed to the lack of precise therapeutic targets for methylprednisolone and its extensive influence on multiple metabolic pathways. Therefore, it is essential to explore the precise molecular targets and key metabolic pathways of methylprednisolone in treating TMG. This will help mitigate or even eliminate adverse drug reactions, achieving precision therapy.

Metabolomics enables comprehensive analysis of metabolites in biological samples, identifying differential metabolites and related metabolic pathways, which can provide biomarkers and mechanistic insights for disease diagnosis and treatment. Network pharmacology predicts drug targets, constructs PPI networks, and performs enrichment analyses to uncover the molecular mechanisms and signaling pathways of drug actions. Combining these two approaches allows an in-depth investigation into the potential mechanisms of methylprednisolone in TMG treatment, offering a scientific foundation for precision therapy.This study utilized untargeted metabolomics based on Liquid Chromatography-Mass Spectrometry (LC—MS) and network pharmacology analyses to identify TNF as a key target and ceramide (Cer) and sphingomyelin (SM) as metabolic biomarkers of methylprednisolone treatment in TMG. The identified key targets and metabolic biomarkers were further validated in an additional 20 post-surgical TMG patients treated with methylprednisolone using enzyme-linked immunosorbent assay (ELISA). This study may provide new therapeutic strategies for TMG patients.

## Methods

### Study subjects

Fifteen newly treated TMG patients from the Myasthenia Gravis Diagnosis and Treatment Center at Shijiazhuang People's Hospital were selected for this study. The cohort included 9 females and 6 males, aged 24–63 years, with a mean age of 44.3 ± 3.1 years. The disease duration ranged from 3 to 36 months, with an average duration of 8.9 ± 2.5 months. All patients underwent extended thymectomy and still had symptoms of myasthenia gravis after surgery. The patients were divided into two groups: the pre-treatment group (TMGPP) and the post-treatment group (TMGPA), based on their status before and after methylprednisolone treatment.Diagnostic criteria: The diagnosis of thymoma was based on the postoperative pathological report. The diagnostic criteria for MG included typical clinical features of MG, positive neostigmine test, positive detection of antibodies such as anti-AChR and MUSK, or a positive electromyography test(repetitive nerve stimulation). Inclusion criteria: clear postoperative pathological diagnosis of thymoma; clear diagnosis of MG; initial treatment after surgery; all patients experienced significant symptom relief after methylprednisolone pulse therapy, with the degree of symptom relief based on the absolute and relative scoring system of MG (ARS-MG) [12]. Exclusion criteria: Type C thymoma (thymic carcinoma); concomitant severe underlying diseases, such as cardiac, liver, or renal dysfunction, which preclude steroid therapy; concomitant other autoimmune diseases, such as systemic lupus erythematosus, rheumatoid arthritis, etc. This study was approved by the Ethics Review Committee of Shijiazhuang People's Hospital (No.[2022]047).The general information of all enrolled patients is shown in Table 1.

**Table 1:** Baseline characteristics of patients

No	Gender	Age	Disease Duration (months)	MGFA Classification	Thymoma Pathology	AchR-ab concentration(Before/After Treatment)	ARS-MG
1	Male	41	4	IIIb	B1/B2	20.01/6.06	89.67%
2	Male	27	12	V	B2	13.18/15.77	77.8%
3	Female	28	5	IIIb	B3	11.96/8.67	94.7%
4	Female	51	3	IIIb	B2	11.05/15.95	100%
5	Male	43	4	V	B1	14.66/11.96	97.5%
6	Female	44	2	IVb	A	2.1/1.29	95.6%
7	Female	54	1	IVa	AB	15.56/5.94	98.3%
8	Male	51	8	IIb	B3	15.38/7.19	95.45%
9	Female	52	15	IIIa	B2	14.0 1/2.87	100%
10	Female	63	25	IIIb	B1	10.57/4.83	91.23%
11	Female	24	1	IIIb	B1	11.49/11.05	100%
12	Male	61	5	IVb	B2	9.21/9.05	98.2%
13	Female	49	36	IIb	AB	13.83/14.8	94.2%
14	Female	45	4	IIIb	B3	18.56/6.18	93.4%
15	Male	32	8	IVb	B2	15.74/15.02	90.04%

MGFA:Myasthenia Gravis Foundation of America; AchR-ab concentration is detected by RIA, with a normal value of < 0.5

### Metabolomics study methods

#### Sample preparation and quality control

Peripheral blood (3 mL) was drawn from all enrolled patients before methylprednisolone pulse therapy and placed in additive-free collection tubes, and again 3 months after the completion of treatment. The collected peripheral blood was transferred to 15 mL centrifuge tubes and left on ice for 1 h, followed by centrifugation for 5 min (x1150 g, room temperature) to separate the serum, which was collected from the upper layer. A 100 μL sample of the serum was then taken and added to 10 μL of methanol-prepared L-2-chlorophenylalanine (concentration 0.3 mg/mL) as an internal standard reference, and vortexed for 15 s using a vortex mixer. Metabolites were extracted using the protein precipitation method: the sample from step 2 was added to 300 μL of acetonitrile-methanol mixture (V:V = 1:2), vortexed for 1 min to precipitate proteins; subjected to ultrasonic extraction for 15 min in an ice-water bath, and then left to stand at −20 °C for 30 min; centrifuged for 8 min (4 °C, x15800 g), and 400 μL of the supernatant was transferred into an LC–MS autosampler vial and dried.The residue was redissolved in 400 μL of a water–methanol mixture (v:v = 4:1)(vortexed for 30 s, ultrasonicated for 3 min). This mixture balances solubility and polarity, effectively dissolving various metabolites. It also provides stability, reducing metabolite degradation and adsorption during extraction and storage; left to stand for 2 h at −20 °C; centrifuged for 8 min (4 °C, x15800 g), and 150 μL of the supernatant was collected with a 2 mL syringe, filtered through a 0.22 μm organic phase pinhole filter, and transferred into an LC injection vial, and stored in a −80 °C freezer until LC—MS analysis. Quality Control (QC) samples were prepared by mixing equal volumes of extracts from all samples, processed according to the same pre-treatment method as the test samples.

#### LC–MS analysis conditions

The following method and conditions were used for liquid chromatography–mass spectrometry (LC-MS) analysis to ensure accuracy and reliability:

Chromatography: An ACQUITY UPLC I-Class system with a VION IMS Q-Tof high-resolution mass spectrometer was used. The ACQUITY UPLC BEH C18 column (100 × 2.1 mm, 1.7 μm) was set at 45 °C. The mobile phase consisted of water with 0.1% formic acid (A) and acetonitrile with 0.1% formic acid (B), with a flow rate of 0.5 mL/min. The gradient elution program was as follows: 0 min (99% A, 1% B), 1 min (70% A, 30% B), 2.5 min (40% A, 60% B), 6.5 min (10% A, 90% B), 8.5 min (0% A, 100% B), 10.7 min (0% A, 100% B), 10.8 min (99% A, 1% B), and 13 min (99% A, 1% B).

Mass Spectrometry: An electrospray ionization (ESI) source was used in positive/negative ion mode. Parameters were set as follows: capillary voltage 2.5 kV, DP voltage 40 V, collision energy (CE) 4 eV, source temperature 115 °C, desolvation temperature 450 °C, desolvation gas flow 900 L/h, m/z range 50–1000 amu, scan time 0.2 s, and scan interval 0.02 s.

#### Data pre-processing

The raw data were processed using the metabolomics software Progenesis QI v2.3, including noise reduction, baseline correction, de-isotoping, peak detection, peak alignment, binning, and QI normalization. Compound identification was based on secondary fragments, accurate mass, and isotopic distribution, using the Human Metabolome Database (HMDB), METLIN database, LipidMaps (v2.3), and an in-house database for qualitative analysis. In the extracted data, ion peaks with missing values (zero values) exceeding 50% within a group were removed. Missing values were replaced with half of the minimum value. Compound selection was based on the qualitative score (Score), with a maximum score of 60, allocated as follows: 20 points for accurate mass deviation, 20 points for secondary fragment matching accuracy, and 20 points for isotopic distribution matching. Compounds with a Score ≥ 36 were considered reliably identified, while those scoring below 36 were regarded as inaccurately identified and excluded. The metabolomics data after preprocessing approached a normal distribution.

#### Data analysis

The MetaboAnalyst software was used to perform Principal Component Analysis (PCA) initially, reducing the high-dimensional information of metabolites and monitoring the stability of the entire analysis process as well as the distinguishability between the two groups of samples. Orthogonal Partial Least Squares Discriminant Analysis (OPLS-DA) with supervision was employed to predict sample categories and filter out noise unrelated to the classification criteria. Through OPLS-DA analysis, each metabolite could obtain a metric, namely the Variable Importance in Projection (VIP). The larger the VIP value, the greater the contribution of the substance to the differentiation between the two groups, making this parameter an important reference indicator for selecting differential metabolites. To prevent overfitting of the OPLS-DA model, a permutation test was used to evaluate the OPLS-DA model. SPSS 25.0 was used for paired sample T-tests on metabolites to compare the differential products between the two groups, with *P* < 0.05 considered statistically significant. The criteria for selecting differential metabolites were VIP > 1 in OPLS-DA analysis and *P* < 0.05 in T-tests. To avoid false positives, the selected differential metabolites were subjected to FDR correction, and metabolites with an FDR > 0.05 were excluded. Pathway enrichment analysis was performed on all identified significant differential metabolites based on the KEGG database.

### Network pharmacology study methods

"Swiss Target Prediction"database was queried with"Methylprednisolone hemisuccinate"to identify all potential targets of methylprednisolone;"Myasthenia Gravis with thymoma"was used as the search term to collect disease targets through databases such as"GeneCards,""DisGeNet,""OMIM,"and"drugbank."The intersection of methylprednisolone's action targets with TMG disease targets was taken to determine the potential targets for the drug's treatment of the disease. The filtered potential targets were then imported into the STRING (https://string-db.org/) protein interaction analysis platform, choosing"Multiple proteins"to enter the intersecting genes and selecting the species as"Homo sapiens"to obtain the Protein–Protein Interaction (PPI) network. A confidence threshold of 0.4 was chosen for further target filtration and data export. The data were imported into Cytoscape 3.7.1, where the"network analyzer"feature was used to determine the degree values of interacting proteins. Node sizes were determined by the degree, and the thickness of edges was determined by the combined score, to construct a PPI network diagram. The Metascape database was utilized for Gene Ontology (GO) analysis and KEGG pathway enrichment analysis of the obtained genes.

### Verification of targets and metabolic biomarkers

#### ELISA experiment procedure

We selected 20 patients who met the previously described inclusion criteria and collected 3 mL of venous blood from each patient before and 3 months after methylprednisolone treatment. The ELISA method was used to validate the upstream synthetic enzymes of the key metabolites, and targets in the TMGPA and TMGPP groups.

During the experiment, standard and sample wells were set up. Standard solutions of different concentrations were added to standard wells. For sample wells, sample diluent and then the test samples were added with appropriate dilution. Next, enzyme-labeled reagent was added to each well (except blank wells) and incubated at 37 °C for 60 min. After incubation, the plate was washed five times with wash solution to remove unbound substances. Substrate A and B were then added and incubated at 37 °C in the dark for 15 min. The reaction was stopped by adding stop solution, turning the solution from blue to yellow. Finally, absorbance (OD value) was measured at 450 nm within 15 min using a multimode microplate reader. Sample concentrations were determined via standard curves and multiplied by the dilution factor for final concentration.

####  Statistical analysis

The data from the experimental validation were statistically analyzed using SPSS 25.0. For variables that followed a normal distribution, paired t-tests were used. For variables that did not follow a normal distribution, non-parametric rank-sum tests were applied.

## Results

### Metabolomics analysis results

#### Metabolic profiling analysis

**Fig. 1:**
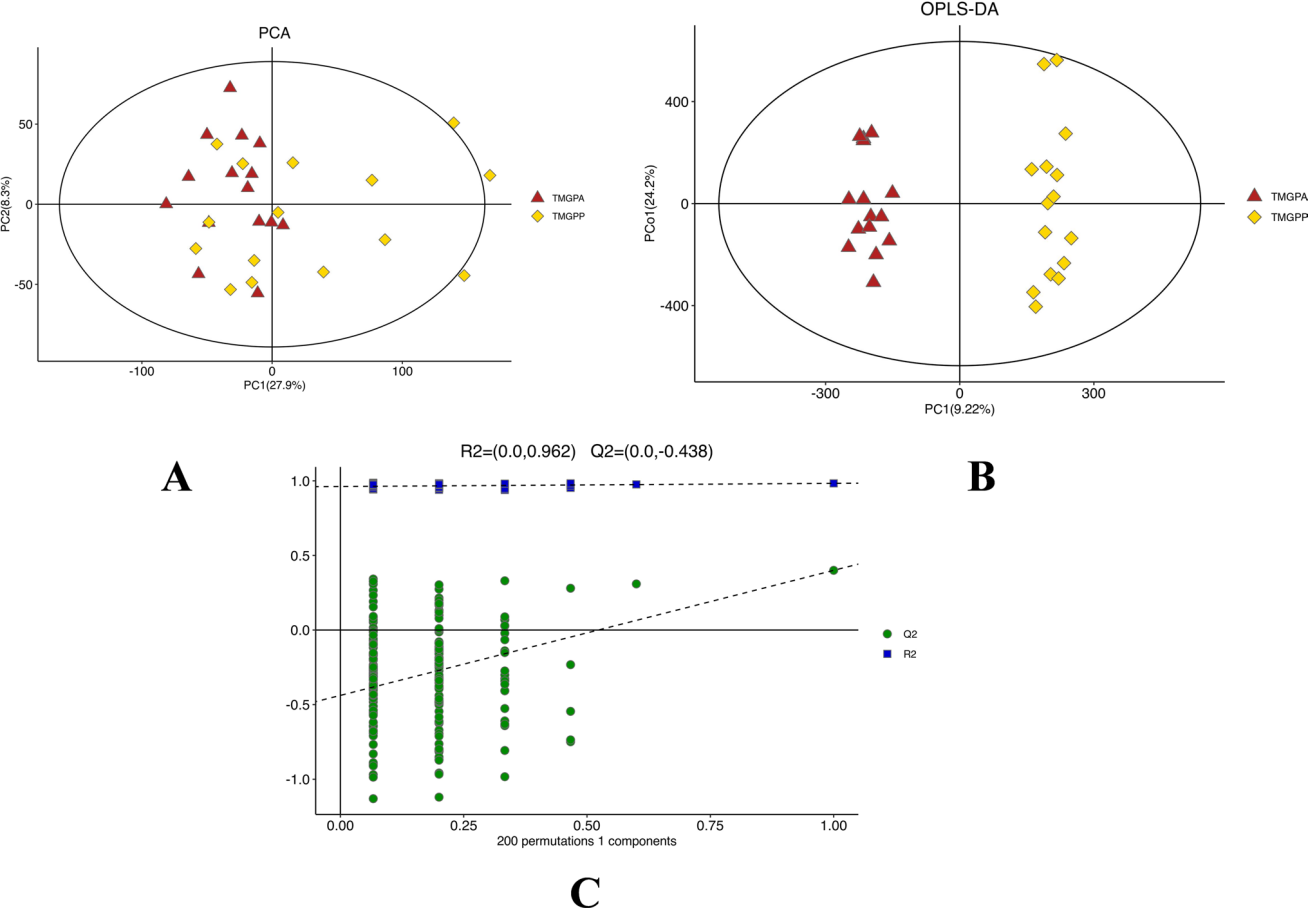
Serum profile analysis results before and after treatment with methylprednisolone in patients with TMG. **A:** PCA score plot shows that there is a trend of separation between the two groups.The elliptical area represents the 95% confidence interval.**B:** OPLS-DA score plot, shows that the two groups can be more clearly separated, indicating significant differences between the groups.**C:** OPLS-DA permutation test plot.The Q2 values on the left side of the figure are all smaller than the Q2 values of the original model on the right side, indicating that the OPLS-DA model simulates well

The PCA score plot indicated an R2X of 0.533, demonstrating the model's reliability. The intra-group variation within the TMGPA was smaller compared to the TMGPP, with a certain trend of separation between the two groups (Fig. 1A). The OPLS-DA analysis results showed complete separation between the two groups of samples, indicating significant inter-group differences (R2X = 0.585, R2Y = 0.983, Q2 = 0.745, Fig. 1B); in the 200 permutations test, the Q2 values of the permutation model were all lower than those of the original model, indicating good predictive power and reliability of the model (Fig. 1C). Therefore, it can be concluded that there were significant changes between the TMGPA and TMGPP groups.

#### Screening of differential metabolites

**Fig. 2:**
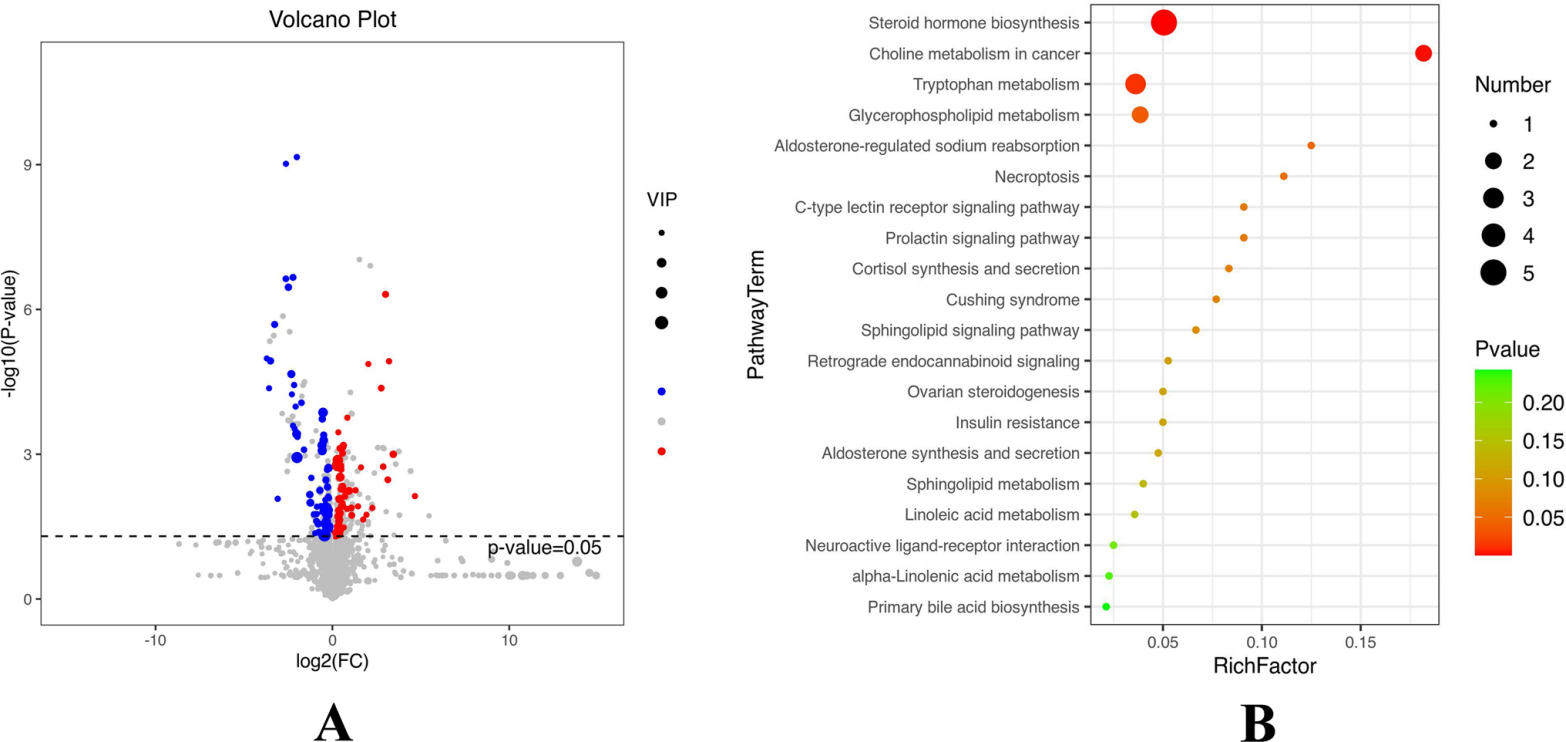
Distribution and functional annotation of differential metabolites. **A:** Volcano plot of differential metabolites; red means up—regulated metabolites, and blue means down—regulated ones **B:** KEGG enrichment analysis of differential metabolites. the larger the Richfactor, the greater the enrichment; the color from green to red indicates that the p-value decreases in order; the larger the dot, the greater the enrichment to the The larger the dot, the greater the number of metabolites enriched to the pathway

By combining OPLS-DA analysis with the t-test, 148 metabolites were found, of which 25 were enriched in KEGG pathways. After FDR correction, 21 metabolites remained significant. There were 5 upregulated metabolites, including LysoPC (LyP), 24-hydroxycholesterol, etc.; and 16 downregulated metabolites, including Ceramide(Cer), sphingomyelin (SM), uric acid, phosphatidylcholines (PC), creatine, L-fucose, among others (Table 2). Volcano plots were used to visualize the p-values, VIP values, and fold change (FC) values (Fig. 2A).

**Table 2:** Bisic information of differential metabolite expression

Metabolites	KEGG	VIP	P**-**value	M/Z	FC	State
11-beta-hydroxyandrosterone-3-glucuronide	C05643	1.406	0.0000	481.244	0.223	Down
Cer(d18:0/16:0)	C00195	1.742	0.0143	540.534	0.899	Down
SM(d18:1/22:0)	C00550	7.482	0.0006	540.330	0.664	Down
SM(d18:1/24:1(15Z))	C00550	7.953	0.0008	588.330	0.670	Down
24-Hydroxycholesterol	C13550	1.548	0.0167	134.060	1.294	UP
3-Methyldioxyindole	C05834	1.261	0.0083	447.347	0.117	Down
Alpha-N-Phenylacetyl-L-glutamine	C04148	2.303	0.0184	181.096	2.131	UP
Androstenedione	C00280	1.941	0.0001	162.055	0.161	Down
Androsterone glucuronide	C11135	1.470	0.0031	445.331	0.438	Down
Creatine	C00300	1.003	0.0432	469.333	0.507	Down
DHEA sulfate	C04555	15.269	0.0307	331.191	0.249	Down
Indole-3-ethanol	C00955	2.816	0.0326	465.249	0.829	Down
L-Acetylcarnitine	C02571	2.896	0.0056	585.270	0.615	Down
L-Fucose	C01019	1.051	0.0118	407.207	0.635	Down
LysoPC(20:5(5Z,8Z,11Z,14Z,17Z))	C04230	4.560	0.0057	367.158	1.919	UP
LysoPC(18:1(11Z))	C04230	10.194	0.0458	204.122	1.131	UP
LysoPC(20:2(11Z,14Z))	C04230	6.561	0.0029	144.101	1.360	UP
PC(22:5(4Z,7Z,10Z,13Z,16Z)/16:1(9Z))	C00157	11.017	0.0391	538.314	0.805	Down
PC(14:0/20:2(11Z,14Z))	C00157	21.748	0.0475	566.345	0.736	Down
Phthalic acid	C01606	1.468	0.0008	586.315	0.761	Down
Uric acid	C00366	4.741	0.0155	544.339	0.846	Down

#### KEGG enrichment analysis of differential metabolites

The differential metabolites were input into the KEGG database for metabolic pathway enrichment analysis, which identified the top 20 ranked metabolic pathways. These included steroid hormone biosynthesis, C-type lectin receptor signaling pathway, choline metabolism in cancer, necroptosis, glycerophospholipid metabolism, insulin resistance, among others. The results were illustrated in a bubble chart (Fig. 2B).

### Network pharmacology analysis results

#### Drug-disease target intersection

**Fig. 3:**
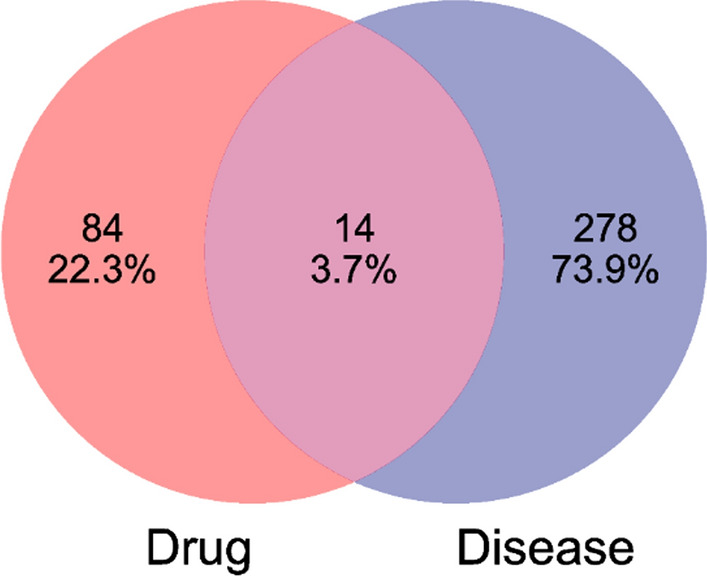
Venn diagram of methylprednisolone and TMG;` 292 disease—related targets and 98 drug—related targets, with 14 overlapping targets between them

**Fig. 4:**
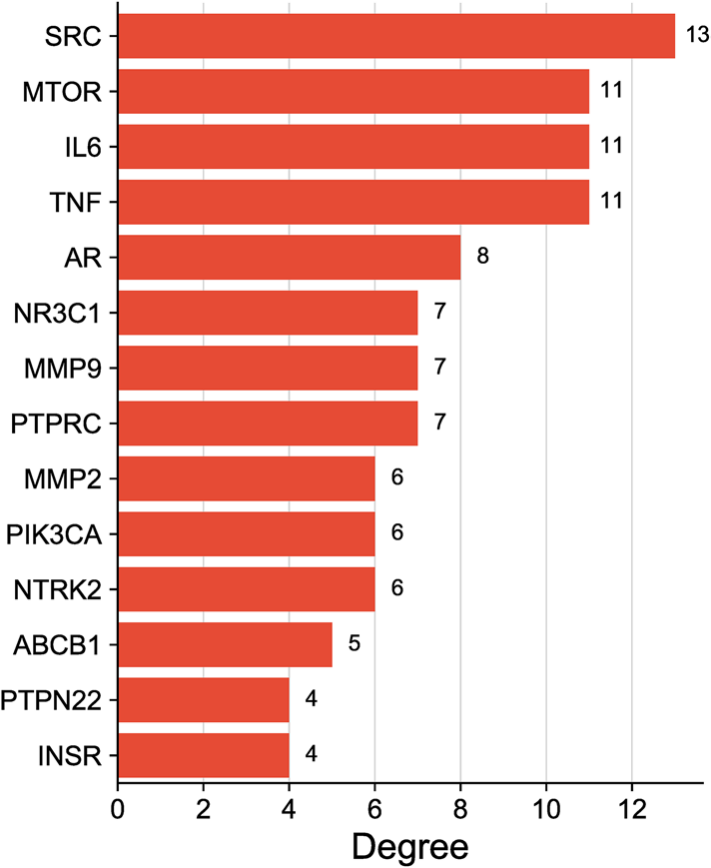
Intersection target degree diagram.The horizontal coordinate represents the degree value and the vertical coordinate represents the target point

A total of 98 targets related to methylprednisolone were obtained from the"Swiss Target Prediction"database. Through databases such as"GeneCards,"a total of 336 disease-related targets were identified, and after removing duplicates, 292 unique targets remained. An intersection of these 292 targets with the previously predicted 98 drug targets yielded 14 intersecting targets (Fig. 3), including ABCB1, NR3 C1, MTOR, SRC, IL-6, TNF, AR, MMP2, PIK3 CA, MMP9, NTRK2, PTPN22, PTPRC, INSR, etc. Protein–Protein Interaction (PPI) network analysis revealed that the top 10 targets by degree ranking included SRC (Degree = 13), MTOR (Degree = 11), IL6 (Degree = 11), TNF (Degree = 11), AR (Degree = 8), NR3 C1 (Degree = 7), MMP9 (Degree = 7), PTPRC (Degree = 7), MMP2 (Degree = 6), PIK3 CA (Degree = 6)(Fig. 4).

#### Enrichment analysis results

**Fig. 5:**
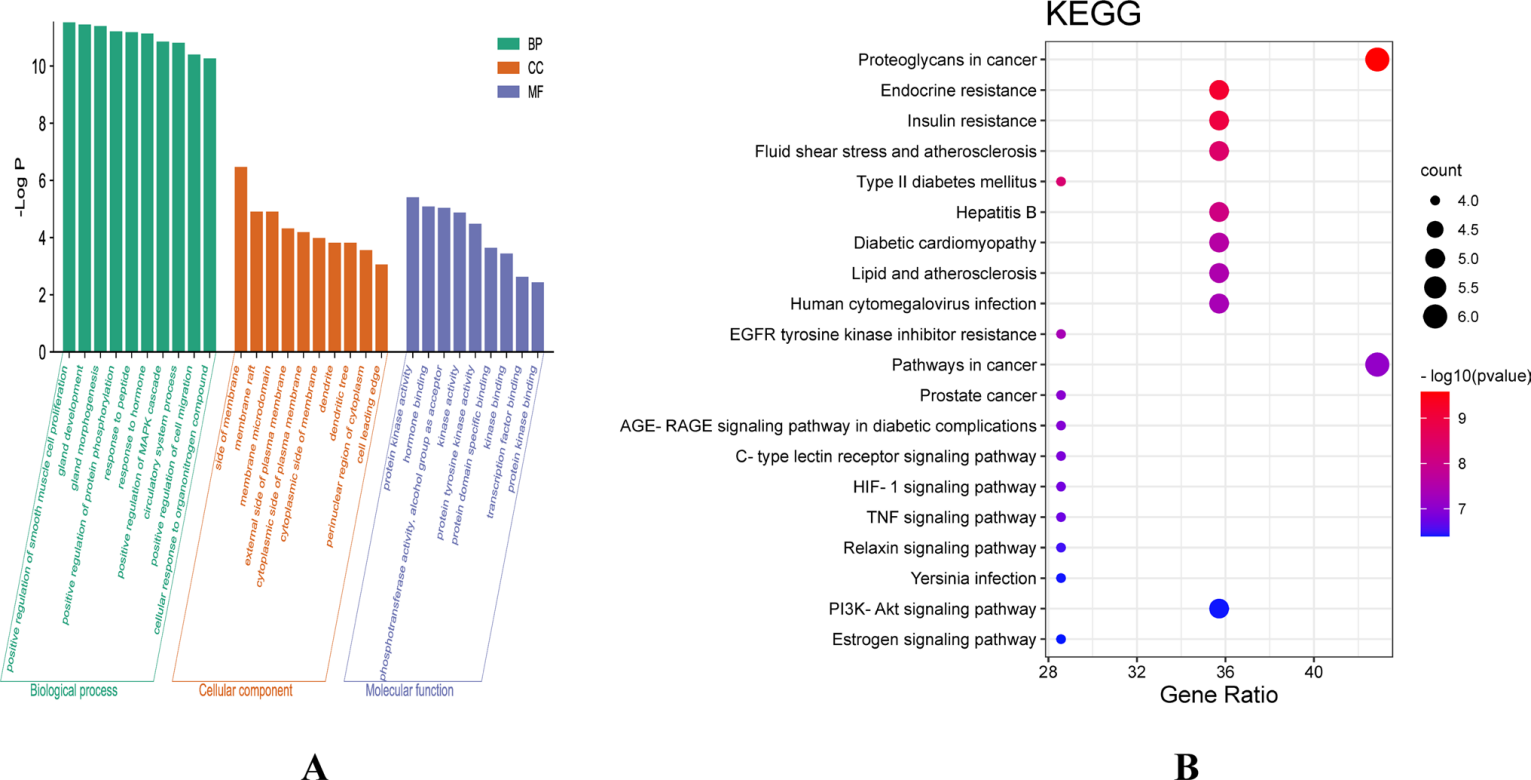
Functional annotation of key targets **A:** GO enrichment analysis diagram.The green part is for biological processes, the red part is for cellular components, and the blue part is for molecular functions. **B:** KEGG enrichment pathway diagram of key targets.The horizontal coordinate is the gene rate; the greater the gene rate, the greater the enrichment; the color from red to blue indicates that the p-value decreases in order; the larger the dot, the greater the number of gene targets enriched to that pathway

The 14 target genes selected based on the PPI were analyzed for enrichment in the DAVID database. The top 10 significant enriched GO functions and KEGG metabolic pathways were identified based on their gene numbers, resulting in 156 GO entries, with 119 under Biological Processes (BP) involving pathways such as positive regulation of smooth muscle cell proliferation and gland development. Molecular Function (MF) was enriched with 21 entries, involving protein kinase activity, hormone binding, etc., and Cellular Component (CC) had 16 entries, involving pathways like the side of the membrane, membrane raft, membrane microdomain, external side of the plasma membrane, cytoplasmic side of the plasma membrane, etc.(Fig. 5A). A total of 76 signaling pathways were selected in the KEGG pathway enrichment analysis, with visualization based on the order of gene numbers, results shown in Fig. 5B. This includes pathways involved in proteoglycans in cancer, lipid and atherosclerosis, endocrine resistance, TNF signaling pathway, among others.

### ELISA validation results

**Fig. 6:**
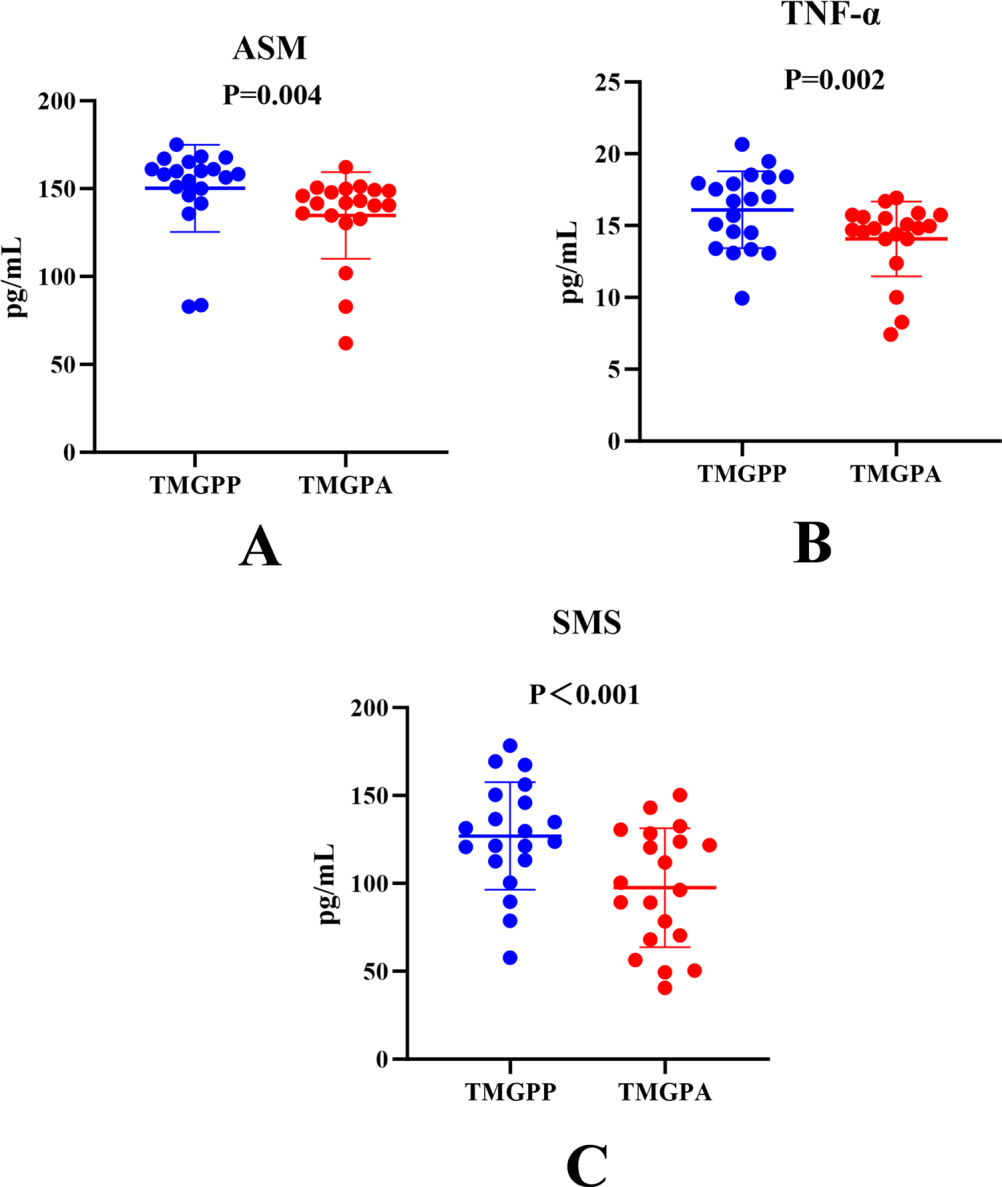
The expression of ASM, TNF-α, and SMS were all downregulated after treatment with methylprednisolone **A:** Expression of ASM **B:** Expression of TNF-α **C:** Expression of SMS

An additional 20 patients who met the aforementioned inclusion criteria were selected, and 3 mL of venous blood was collected from each patient before methylprednisolone treatment and again 3 months after treatment. The ELISA method was used to validate the key metabolic enzymes and target of the TMGPA and the TMGPP. The statistical analysis showed that ASM, SMS, and TNF-α all showed a downward trend after methylprednisolone pulse therapy (Fig. 6), and the differences were statistically significant (*P* < 0.05).

## Discussion

Methylprednisolone, a commonly used glucocorticoid in clinical practice, has shown significant clinical efficacy in patients with TMG [13], yet its specific molecular mechanisms have not been fully elucidated. Our research has discovered that methylprednisolone is a multi-target drug. Due to its non-specific targeting, it can exert a variety of pharmacological effects, which also leads to numerous adverse reactions during treatment. This study employed metabolomics combined with network pharmacology analysis to elucidate the potential targets and metabolic biomarkers of methylprednisolone treatment for TMG at the genetic and metabolite levels.

Metabolomics analysis revealed that differential metabolites such as ceramide and sphingomyelin might be related to the treatment of TMG with methylprednisolone, involving metabolic pathways like necroptotic death pathway, C-type lectin receptor signaling pathway, and glycerophospholipid metabolism. SM is an essential component of cell membrane lipoproteins in the human body, mainly composed of ceramide and phosphocholine, playing a vital physiological role in cell homeostasis [14].Cer is a central molecule in the sphingomyelin signaling pathway, produced by the hydrolysis of SM by acid sphingomyelinase (ASM), acting as a second messenger. Research has shown that Cer participates in activating various protein kinases and phosphatases, thereby initiating downstream signaling pathways, inducing the expression of various inflammatory proteins, amplifying the cascade of inflammation, and regulating physiological functions such as cell apoptosis, stress, immunity, and inflammation [15].

Cer plays a critical role in both innate and adaptive immune responses [16]. In the innate immune response, pathogen-associated molecular patterns (PAMPs) can activate Toll-like receptors (TLRs) on the membrane of monocytes/macrophages, causing oxidative stress and thereby activating ASM to increase Cer production, which induces the production of inflammatory factors such as IL-6 [17]. In the adaptive immune response, Cer can participate in the differentiation and function regulation of T cells/B cells. Due to Cer's significant role in both innate and adaptive immune responses, it is involved in the pathogenesis of various inflammatory diseases and autoimmune diseases. Studies by Miltenberger- Miltenyi et al found that Cer levels were significantly increased in patients with rheumatoid arthritis compared to healthy controls [18]. Animal experiments have shown that pharmacological inhibition or genetic knockout of ASM can reduce arthritis symptoms and pro-inflammatory cytokines in mouse joints [19]. Checa's research proved that sphingomyelin metabolism is dysregulated in systemic lupus erythematosus (SLE) patients, and this dysregulation is closely related to disease activity [20]. In their study, Cer (C16:0) levels were significantly higher in SLE patients than in healthy individuals, but returned to normal after immunosuppressive treatment. In studies on Kawasaki Disease (KD), comparing ASM levels between KD patients and control groups revealed that the former's levels were higher than the latter, indicating ASM's involvement in the pathogenesis of KD [21].

Studies have demonstrated that Cer may play a crucial role in the pathogenesis of MG and serve as a novel potential biomarker for disease severity [22]. A study with a cross—sectional design recruited 73 MG patients and 52 healthy controls. It revealed that four plasma ceramides (C16:0—Cer, C18:0—Cer, C24:0—Cer, C24:1—Cer) in MG patients were significantly elevated compared to healthy controls and correlated positively with disease severity. Additionally, C18:0—Cer correlated positively with serum pro—inflammatory cytokines (IL—1β, IL—6, IL— 17 A, IL—21) and the proportion of circulating memory B cells, suggesting it may contribute to MG pathogenesis by modulating the Th17/Treg balance, promoting inflammation, and activating B cells. As an autoimmune disease, MG causes the production of a large number of autoantibodies in the body, involving the activation of various immune cells such as lymphocytes and inflammatory responses. Our metabolomics study found that the production of Cer decreases after treatment with methylprednisolone, thereby inhibiting a series of biological activities produced by Cer, acting as an immunosuppressant.

Recent studies have also found that sphingomyelin metabolism plays an important role in the occurrence and development of tumors. Sphingomyelin is an essential component of mammalian cells, and cells lacking SM synthesis exhibit proliferation disorders [23]. Sphingomyelin promotes tumor proliferation and metastasis, with an increased content of sphingomyelin in various tumor cells and the expression of its key synthetic enzyme, sphingomyelin synthase (SMS), also increased. Studies have shown that increased expression of SM and SMS in breast and ovarian cancers may facilitate tumor metastasis [24, 25]. Further research indicated that the oncogene BCR-ABL, responsible for chronic myeloid leukemia (CML), has been shown to upregulate the expression and activity of SMS in CML cell lines [26]. Disrupting sphingomyelin metabolism or depleting SMS can inhibit the growth and migration of ovarian cancer cell lines and suppress the formation of the tumor microenvironment (TME) [27]. We hypothesize that SM similarly promotes the growth of thymoma and the generation of TME within thymoma. Even after thymectomy, the TME may still persist in the body, and the downregulation of SM following methylprednisolone pulse therapy may inhibit TME, thereby reducing thymoma recurrence.

In network pharmacology research, GO and KEGG enrichment analyses revealed that the potential mechanisms of methylprednisolone treatment for TMG mainly focus on proteoglycan-related cancer pathways and tumor necrosis factor-alpha (TNF-α) pathways, involving therapeutic targets such as tumor necrosis factor (TNF) and interleukin-6 (IL-6). TNF is a pleiotropic cytokine produced by activated macrophages and other immune cell types, such as T lymphocytes, NK cells, and neutrophils, including TNF-α and TNF-β, with TNF-α playing a key role in the regulation of immunity and inflammation [28]. TNF-α exerts multiple biological functions by binding to tumor necrosis factor receptors (TNFR) on the cell membrane, whose activation can further activate downstream inflammatory signaling pathways. TNF-α plays a crucial role in the progression of various autoimmune diseases, with a substantial body of literature reporting increased expression of TNF-α in several autoimmune diseases, and it is also involved in the pathogenesis of MG [29, 30]. Many tumor cells can also increase the expression of TNF-α, further leading to the release of various inflammatory mediators, suppression of T-cell anti-tumor activity, and angiogenesis, thereby inducing tumor cell proliferation and metastasis [31].

**Fig. 7:**
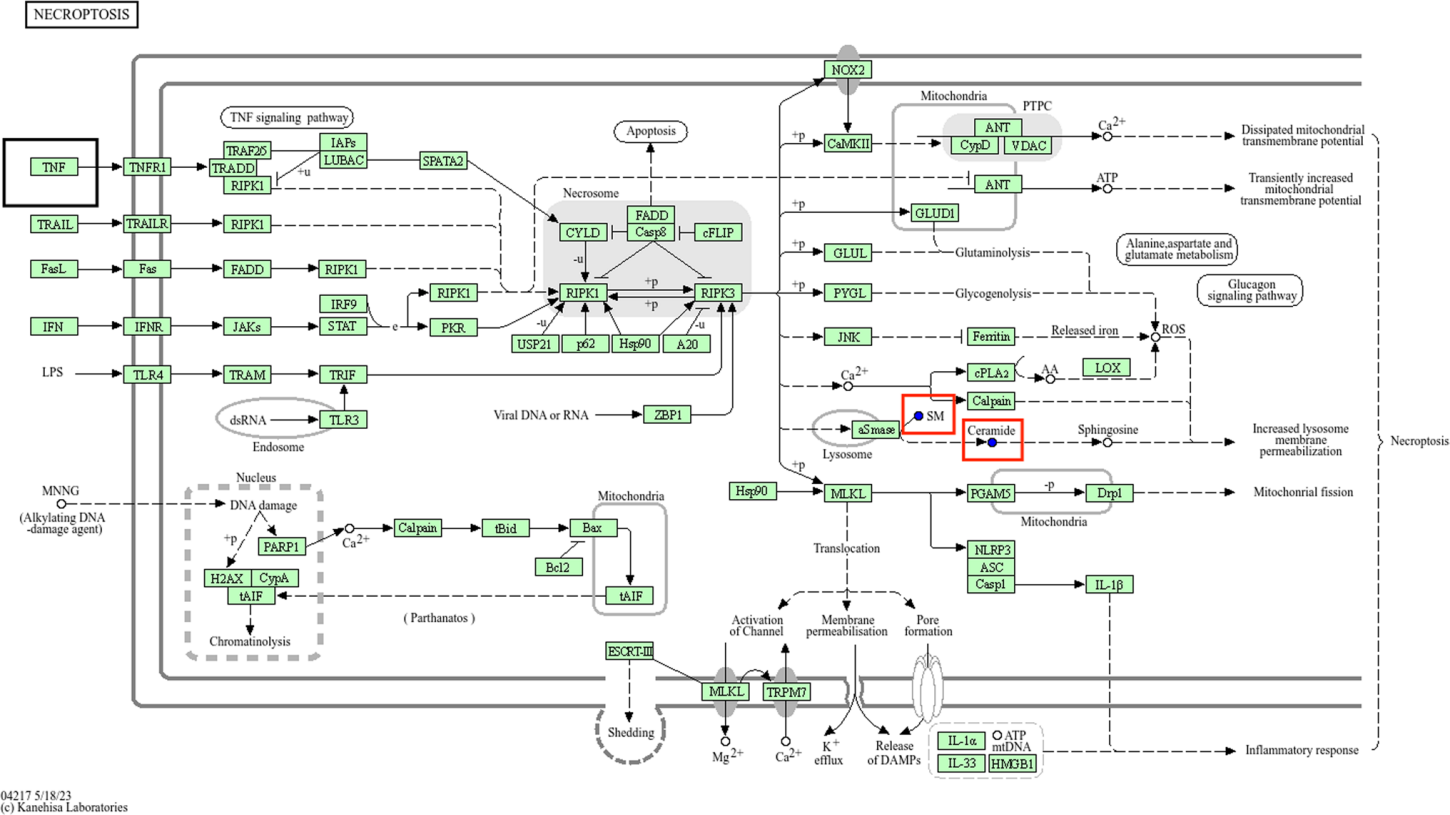
TNF, along with SM and Cer, are co-enriched in the necroptosis pathway, which includes the tumor necrosis factor pathway. Key metabolites are indicated by red boxes, and key targets are marked with black boxes

**Fig. 8:**
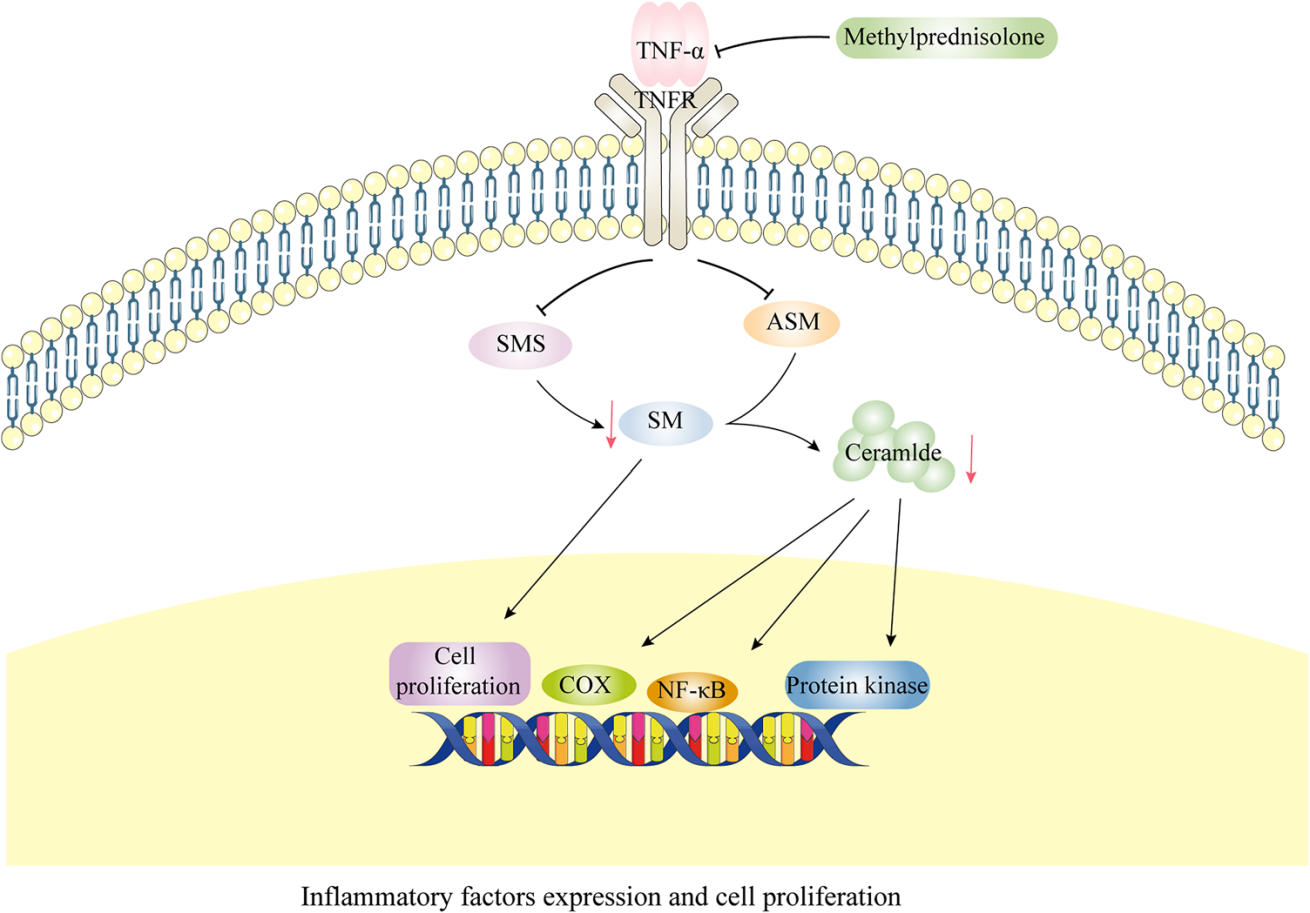
Potential Mechanisms of Methylprednisolone Treatment for TMG

Human life activities cannot be separated from the collective participation of genes, proteins, and various metabolites. Metabolites, being downstream in the regulatory chain, often reflect disturbances at the gene and protein levels, and changes in metabolites can also trigger changes at the genetic level. This study combined serum metabolomics and network pharmacology analysis to find intersections in the pathways obtained from KEGG enrichment analysis. Key metabolites Cer and SM, along with key target TNF, were enriched in the necroptosis pathway (Fig. 7), primarily functioning in the TNF signaling pathway, where the upstream regulatory enzyme ASM can hydrolyze SM to produce Cer. Studies have shown that cytokines such as TNF-α can activate ASM, thereby hydrolyzing SM to produce Cer [32], affecting SMS as well, thus forming a"gene target-pathway-enzyme-metabolite"regulatory network. Methylprednisolone may affect the expression of the TNF gene through the necroptosis pathway, further impacting enzymes that produce Cer and SM, ultimately leading to a decrease in Cer and SM, exerting immunosuppressive and tumor-suppressive effects (Fig. 8). Our subsequent ELISA experiments also validated that ASM and SMS, along with TNF-α, are significantly downregulated after methylprednisolone treatment, proving that ASM and SMS play a key role in linking the TNF target with sphingomyelin metabolism.

In summary, we identified the potential key targets TNF and key metabolites SM, Cer in the treatment of TMG with methylprednisolone, providing a basis for precise clinical treatment. In fact, targeted treatment against TNF-α has been initiated in various autoimmune diseases, such as TNF-α inhibitors significantly improving clinical symptoms in inflammatory bowel disease, rheumatoid arthritis, and psoriasis [33–35]. Additionally, literature studies have shown that targeting TNF-α and TNFR can significantly inhibit the growth of various tumors [36, 37]. Treatments targeting sphingomyelin metabolism have also been reported. For example, studies on the key metabolic enzyme ASM have found that various tricyclic antidepressants, such as desipramine and amitriptyline, can act as functional inhibitors of ASM, becoming a new treatment strategy for inflammatory bowel disease and multiple sclerosis [38]. Recent research has found that blocking the synthesis of SM can enhance the immune response to hepatocellular carcinoma, lymphoma, and glioblastoma [39–41]. The phospholipid analogue miltefosine has been approved for treating metastatic breast cancer and can significantly inhibit SM biosynthesis in liver cancer and other tumor cells, promoting the efflux of cholesterol and SM from the cell membrane [42]. We look forward to similar biologics being used in the treatment of TMG in the future to better benefit patients. Although this study preliminarily explored the mechanisms of methylprednisolone treatment for TMG, it has certain limitations, such as a small sample size. Future research will need to increase the sample size and conduct further validation at the cellular and animal levels.

## Conclusion

Our findings preliminarily explored the potential mechanisms of methylprednisolone treatment for TMG through metabolomics and network pharmacology analysis. The study found that TNF could serve as a potential therapeutic target for methylprednisolone in treating TMG, while Cer and SM could act as potential metabolic biomarkers for evaluating its therapeutic effects, thus laying a foundation for future research into the mechanisms of glucocorticoid treatment for TMG. However, the study has certain limitations, such as a small sample size. Future research will need to increase the sample size and conduct further validation at the cellular and animal levels.

## Data Availability

The raw data supporting the conclusions of this article will be made available by the authors, without undue reservation.
